# The Invisible Web: Linking Diet and Gut Microbiota Across Time and Space in Odonates

**DOI:** 10.1007/s00248-026-02850-y

**Published:** 2026-08-04

**Authors:** K M Kaunisto, N Smolander, M Tamminen, R Stoks, S Kylänpää, I E Sääksjärvi, E J Vesterinen

**Affiliations:** 1 Department of Biodiversity Sciences, University of Turku, Turku, FI-20014 Finland; 2 Department of Biology, University of Turku, Turku, Finland; 3 Evolutionary Stress Ecology and Ecotoxicology, University of Leuven, Leuven, Belgium; 4 Turku Bioscience Centre, University of Turku and Åbo Akademi University, Turku, Finland

**Keywords:** Insect, Microbes, Metabarcoding, Faecal DNA, Damselfly, Bacterial assemblages

## Abstract

The complex relationship between insects and their gut microbiota has become a central theme in host–microbe ecology, revealing profound effects on host fitness and interactions. Yet, how gut microbial communities—and their primary determinant, diet—vary across space and time in natural insect populations remains largely unexplored. Understanding these dynamics is essential for linking microbial variation with host biology. We investigated bacterial gut microbiota and dietary patterns in two odonate species, *Sympetrum vulgatum* and *Lestes sponsa*, across their flight season and three locations in southern Finland. Using faecal DNA metabarcoding of the cytochrome c oxidase subunit I (COI) gene for diet and 16 S rRNA gene for gut microbiota, we examined spatiotemporal changes and their associations. Season, location, and host species affected both dietary and bacterial diversity. The observed parallel responses of dietary composition and microbial communities highlight that the same ecological factors shaping insect feeding behaviour also influence their associated microbiota, illustrating the dynamic and context-dependent nature of insect–microbe interactions in the wild and emphasizing the need for broader ecological perspectives in microbiome research.

**Supplementary Information:**

The online version contains supplementary material available at https://doi.org/10.1007/s00248-026-02850-y.

## Introduction

Bacteria are ubiquitous and essential components of ecosystems on Earth, significantly contributing to species diversity, genetic variation, and ecological interactions [1, 2]. As symbionts, bacteria reside within animals, where they may outnumber the host cells by several folds [3, 4]. In the guts of insects, symbiotic bacteria play critical roles in multiple fitness-related traits including host development, foraging, fecundity, and survival (e.g [5, 6]). For example, gut microbiota of insects has been linked to increased oviposition in dipterans [6], heat adaptation in odonates [7], as well as metabolization of environmentally-harmful chemicals in lepidopterans [8]. Furthermore, mosquitoes require a diverse gut microbiota to develop properly [9], and gut microbiota may facilitate ecological niche shifts in insects [10]. These findings emphasize the intimate link between the evolutionary success of insects and their vital gut microbiota.

The significance of insects and their associated microbes in sustaining life on Earth, including human food security, economic stability, healthcare, and overall well-being [11, 12], has led to increased research on insect-host microbiota. While this field has expanded rapidly, previous studies have often overlooked key aspects of spatiotemporal dynamics in insect gut microbiota. For example, geographical variation in insect gut microbiota is still poorly explored, even though it could provide important insights into global host–microbe coevolution and into how climatic differences in host traits may shape evolutionary responses to environmental change [13]. Secondly, there are relatively few studies examining temporal variation in the gut microbiota of wild insects (but see e.g. [14]), . Thirdly, most previous research has focused on lab‑reared species, which may inadequately represent the microbial diversity present in natural populations [15].

To address these limitations, emerging evidence has started to identify key environmental drivers of spatiotemporal variation in insect gut microbiota, including temperature gradients [7], exposure to pesticides and other xenobiotics [16, 17], and dietary composition [18, 19]. Nevertheless, important gaps remain in understanding how these processes differ among species and across geographic scales.

In this study, we investigated spatiotemporal variation in dietary composition and bacterial gut microbiota dynamics in two odonate species: the dragonfly Moustached Darter *Sympetrum vulgatum* (Linnaeus, 1758), representing the suborder Anisoptera, and the damselfly Common Spreadwing *Lestes sponsa* (Hansemann, 1823), representing the suborder Zygoptera. Both focal species occur syntopically, sharing habitats with identical prey availability. Odonates are widely recognized as exemplary model organisms for ecological and evolutionary research due to their ecological tractability, ease of observation, and adaptability to field and laboratory studies (e.g [20]), making them highly suitable models for this research.

Our study addresses the following key questions: *i*) How is the dietary composition associated with the gut microbiota within the two focal odonate species, and *ii*) to what extent do period in the flight season, geographical location, host species, and host sex affect the diet and gut microbiota composition? This study is among the first to examine these relationships in natural insect populations, contributing valuable background information into the ecological dynamics shaping host–microbe interactions.

## Methods

The two target species, *Sympetrum vulgatum* and *Lestes sponsa*, were selected based on pilot surveys, which identified them as the most common odonate species at the study sites. We collected faecal samples from both species at two locations in Southern Finland: Järvelä (WGS84 lat: 60° 27’ 45,886”, lon: 22° 22’ 34,625” and Qvidja (60° 17’ 15,024”, 22° 23’ 4,376”). Additionally, we sampled *S. vulgatum* from a third location, Friskala (60° 23’ 37,038”, 22° 14’ 41,884”), where *L. sponsa* was not present. These sampling sites were chosen to encompass diverse habitats, capturing a broad range of environmental variation in diet. Our first study location, Järvelä, is a rather recently established suburban wetland that was created in 2009 by blocking the drainage system of a former crop field. The second location Qvidja is one of the biodiversity hot spots of Southern Finland, characterized as a more urbanized nutrient-rich wetland. The third location Friskala is a brackish water bay of the Baltic Sea. All sites lie within 25 km of each other under comparable climatic conditions, ensuring environmental coherence while maintaining distinct ecological profiles.

We conducted sampling at two time points during the 2022 flight season. The early part of the flight season, when the adult individuals suitable for handling are the most abundant, occurred from 24 to 26 July, during which we collected 189 individuals. The late flight season sampling took place from 11 to 13 August, during which we collected 209 individuals from the same locations. In total, we sampled 398 individuals (18–22 males and females from both species at 2–3 locations in both periods) for faecal molecular analysis. Only sexually mature individuals with adult colours and hardened wings were included in the study. After catching, each odonate was placed in a sterile 10-ml collection tube containing a piece of dampened paper towel to minimize the risk of desiccation. Following established protocols, damselflies were kept in the tubes for 24 h to facilitate defecation, a duration deemed sufficient for this process [19]. Once the individuals had defecated, we froze the sample. Subsequently, all faecal material was collected from the tubes using sterile forceps and frozen in separate 15-ml Falcon tubes at − 64 °C for further processing and analyses.

### Sample Processing and Molecular Analyses

Methods are described here in brief; a detailed account is provided in the Supplementary Material. To characterize the bacterial assemblages and dietary composition of the focal species, we used established metabarcoding protocols for odonates building on our earlier optimization [18, 21]. In short, individual samples of odonate faeces were processed separately to capture individual variation. DNA was extracted using the NucleoSpin Tissue XS Kit. For comprehensive taxonomic coverage, two primer pairs were used for the amplification of the mitochondrial cytochrome c oxidase subunit I (COI) gene: ANML primers LCO1490 [22] and CO1-CFMRa [23] targeting the first half of the animal COI gene and Leray primers mlCOIintF [24] and jgHCO2198 [25] targeting the latter part of the gene. The microbiota was characterized by amplifying the v4 region of the bacterial 16 S rRNA gene using primers 515 F-Parada and 806R-Apprill [26, 27].

Sequencing was performed on two Illumina MiSeq v3 PE 2 × 300 bp runs by the Finnish Functional Genomics Centre, Turku Bioscience, University of Turku and Åbo Akademi University, Turku, Finland. Sequencing yielded the following numbers of paired-end reads: ANML (5,259,261), Leray (11,028,398), and Bac16S (9,332,092). Labelled raw FASTQ reads, ZOTUs, ZOTU assignations, and ZOTU tables are available in the Dryad Digital Repository under the title: The Invisible Web: Linking Diet and Gut Microbiota Across Time and Space in Odonates, and under https://doi.org/10.5061/dryad.wpzgmsc21.

### Read Processing

Prior to the processing, the ANML and Leray reads were combined. The reads were merged and trimmed for quality using 64-bit VSEARCH version 2.14.2 [28], and the Bac16S reads were truncated to 220 bp. Primers were removed from the merged reads using software cutadapt version 1.14 [29]. Singletons were removed and reads were denoised and chimeras removed using USEARCH version 11 [30], resulting in zero-radius OTUs (ZOTUs). The ZOTUs were mapped back to the original trimmed reads to establish the total number of reads in each sample using VSEARCH. We were able to map 5,024,908 of 5,137,746 (97.80%) of ANML reads, 7,817,794 of 7,875,296 (99.27%) of Leray reads, and 8,771,659 of 9,153,656 (95.83%) of Bac16S reads to our original samples. The ANML and Leray ZOTUs were assigned to taxa using BOLD Systems database with a custom API script [33, 34]. The similarity thresholds for assigned identification varied according to taxonomic level: species 98%, genus 96%, family 90%, order 85%, class 80%, and phylum 70%. For the assignation of Bac16S ZOTUs the database ‘16S RDP training set v18’ (21k seqs, drive5.com/usearch/manual/sintax_downloads.html) and SINTAX probabilistic algorithm implemented in VSEARCH [35] were used. For the chosen 16 S database, the genus level is the lowest taxonomic level. The probability thresholds for assigned identification varied according to taxonomic level: species 90%, genus 85%, family 80%, order, class, phylum, and domain 80%. The data was further filtered to remove artefacts, spurious reads, and nontargets based on information on the numerous control samples, technical replicates, and taxonomy. We removed all the taxa outside Phylum Arthropoda (COI reads) or domains Bacteria or Archaea, as well as Class Chloroplast (Bac 16 S reads). Finally, very rare occurrences (sequence count < 2) were removed resulting in 5,156,086 COI reads and 8,316,871 Bac16S reads.

### Statistical Analyses

The statistical analyses of the data were performed using R v. 4.2.3 and Rstudio [31, 32] on total sum scaled data. Dufrene-Legendre Indicator Species Analysis for both the prey and the bacterial data was performed using R package *labdsv* v.2.1-0 [33] at the genus-level (where possible) and the *p*-values were adjusted using the Holm correction. The alpha diversity (Shannon diversity) of the prey was assessed at genus-level and bacterial samples at ZOTU-level using R package *vegan* v.2.6-4 [34]. The significance of the differences between groups was assessed with two-sided Wilcoxon rank-sum test with Holm multiple comparison correction using the packages *ggpubr* v.0.6.0 [35] and *rstatix* v.0.7.2 [36]. The variances were assessed with Levene’s test using the package *car* v.3.1-2 [37]. To test for a correlation between dietary and gut bacterial Shannon diversity across all samples, we performed Spearman rank correlation test.

To assess the effect of environmental variables on odonate prey and gut microbiota community composition, constrained analysis of principal coordinates (CAP) and partial CAP were performed using the package *vegan* v.2.6-4. The significance of the results was tested using ANOVA like permutation test for CAP with 999 permutations. To identify the odonate prey with a significant effect on the gut bacterial community compositions, a model for CAP was automatically built using a stepwise procedure based on *p*-values. The significance of the CAP results was assessed using ANOVA like permutation test for CAP. We assessed concordance between prey and gut microbiome beta diversity using Procrustes analysis using the package *vegan*. Lingoes-corrected principal coordinate ordinations of Bray–Curtis dissimilarities were compared using the permutation-based method *protest()* with 999 permutations.

## Results

### Prey and Bacterial Diversity among the Odonates

**Fig. 1:**
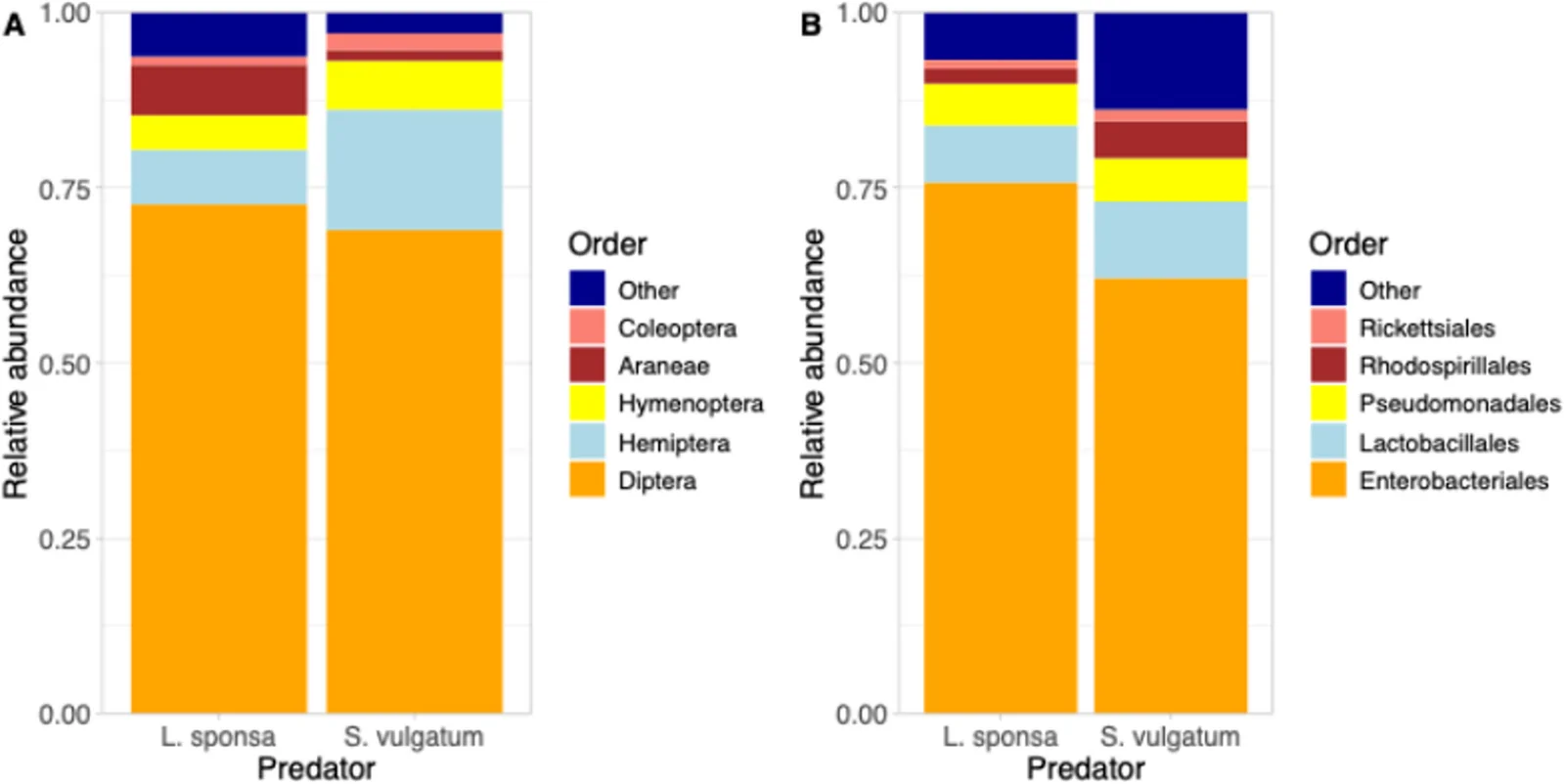
The five most common (**A**) prey and (**B**) bacterial orders in the odonate species L. sponsa and S. vulgatum and their relative abundances, calculated as the proportion of sequencing reads assigned to the order relative to the total reads in each sample

**Fig. 2:**
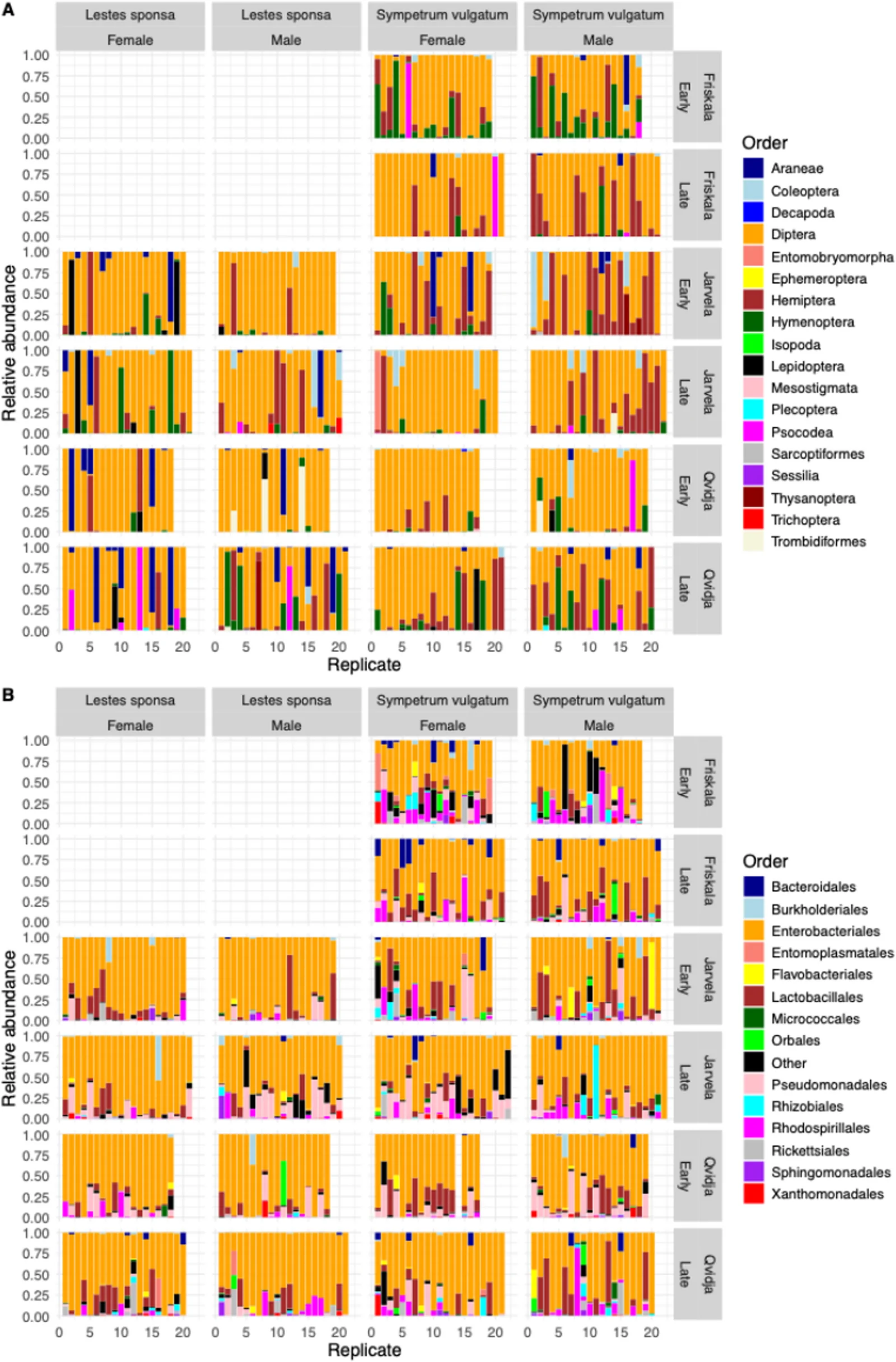
Relative abundances of (**a**) prey arthropod orders and (**b**) bacterial orders in each sample in different odonate species and sexes and sampling sites and seasons, based on the proportion of sequencing reads assigned to the order relative to the total reads in each sample. The 14 most abundant bacterial orders are presented. Taxa outside the 14 most abundant orders or without an order are included as “Other”

The most common prey order across all samples was Diptera (Fig. 1). Some variation was seen among individual odonates as well as between the two odonate (from here on: predator) species, sites, and seasons (Fig. 2a). Hemiptera was the second most common prey order, especially abundant in male odonates of both species in Järvelä as well as in *S. vulgatum* females in Qvidja in both sampling seasons. Other common orders included Hymenoptera, Araneae, Coleoptera, Lepidoptera and Trombidiformes. Trombidiformes are common ectoparasites of many arthropod prey species of odonates, which may explain their abundance likely through secondary predation.

The most common bacterial order across all the sample types was Enterobacteriales (Fig. 1). The second most common order in most of the samples was either Lactobacillales or Pseudomonadales. However, there was again some variation between individual odonates and among sample types (Fig. 2b).

At the family level, *Enterobacteriaceae* was present in all samples with at least 1% relative abundance. The next most prevalent families in *S. vulgatum* were *Acetobacteraceae* and *Pseudomonadaceae* with 38% prevalence each and in *L. sponsa Pseudomonadaceae* and *Enterococcaceae*, with 34% and 30% prevalence, respectively. At ZOTU-level, ZOTU1, the only ZOTU belonging to the genus *Klebsiella* (Family *Enterobacteriaceae*), was present in 76% of the samples and found in all predators, sites, seasons and sexes with at least 1% relative abundance.

### Indicator Species Analysis

We conducted an indicator species analysis to find the prey and bacterial genera that were associated with the two odonate species in different sampling sites and seasons (Supplementary material, Table 1). The prey genus *Anoecia* (a genus of aphids) was significantly associated with *S. vulgatum* (indicator value (indval) 0.379, *p* = 0.046). When comparing seasons, the Diptera *Microchironomus* was associated with the early sampling season (indval 0.392, *p* = 0.046) (several taxa shared the same minimum p-value of 0.0001, which led to identical adjusted p-values of *p* = 0.046 after multiple testing and correction). Comparing the odonate species, site and season combinations revealed that *Lasius* (a genus of ants) was associated with *S. vulgatum* in early Friskala (indval = 0.313, *p* = 0.046). Also, the diptera *Bryophaenocladius* was associated with the *S. vulgatum* from early Qvidja and *Sepsis* with the female *S. vulgatum* from early Friskala (indval = 0.398, *p* = 0.046; indval = 0.300, *p* = 0.046, respectively). The bacterial genera *Asaia* was significantly associated with Friskala (indval = 0.405, *p* = 0.028) and *Ferruginibacter* with early sampling season *S. vulgatum* of Friskala (indval = 0.371, *p* = 0.028) (Supplementary material, Table 2).

### Prey Alpha-diversity Responses between the Odonate Species, Sampling Sites (Locations) and Seasons

**Fig. 3:**
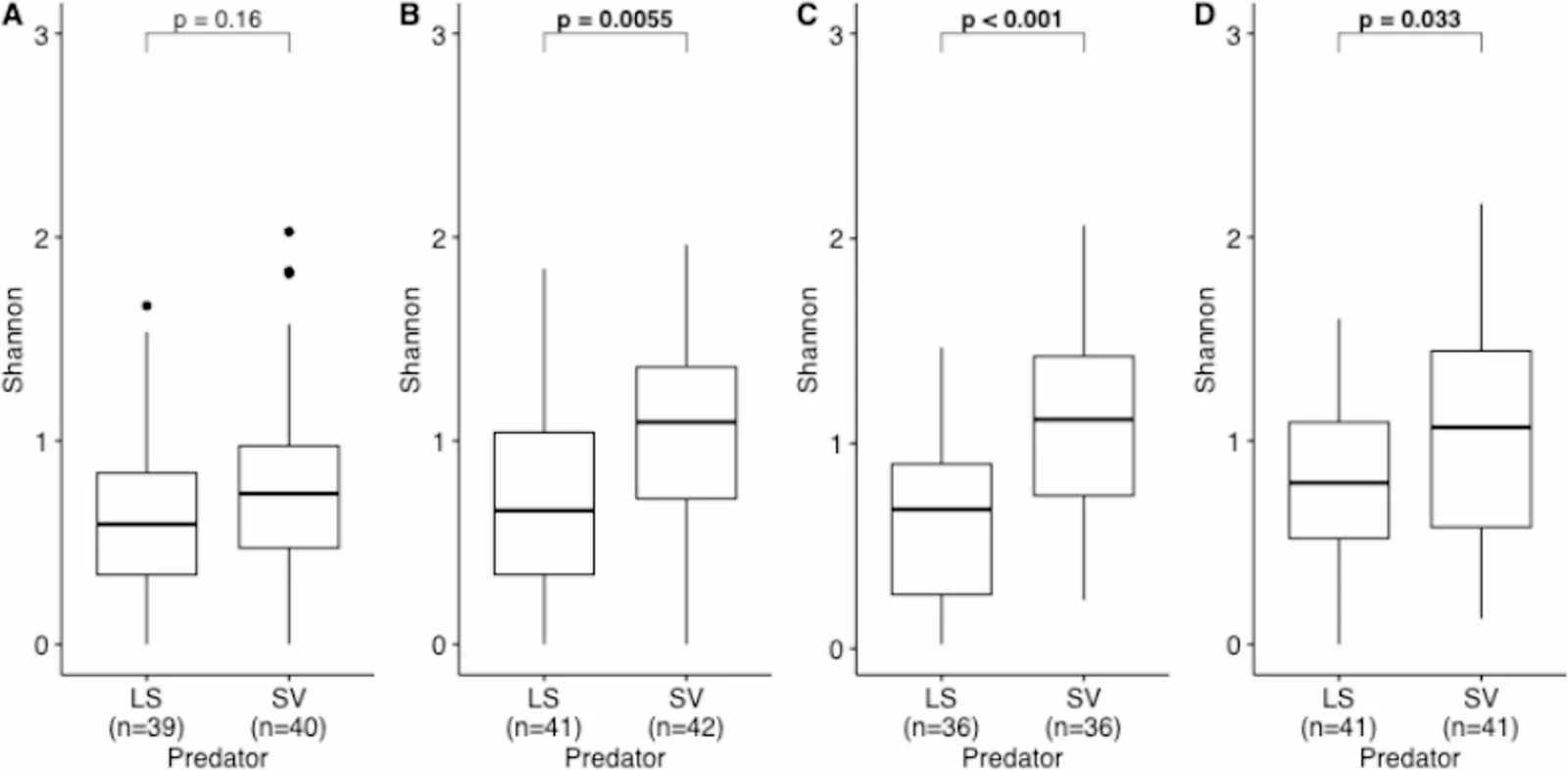
Comparison of prey Shannon diversity indices at genus-level between L. sponsa and S. vulgatum. Boxplots show diversity in (**a**) Järvelä early season, (**b**) Järvelä late season, (**c**) Qvidja early season, and (**d**) Qvidja late season. Statistically significant Holm-adjusted p-values are in bold

To evaluate differences in dietary diversity, we compared the prey Shannon diversity between *S. vulgatum* and *L. sponsa* across different sites and sampling seasons. In both Järvelä and Qvidja, the Shannon diversity was higher in *S. vulgatum* than in *L. sponsa*, in both early and late flying seasons, although the difference was not statistically significant in early Järvelä (Fig. 3).

**Fig. 4:**
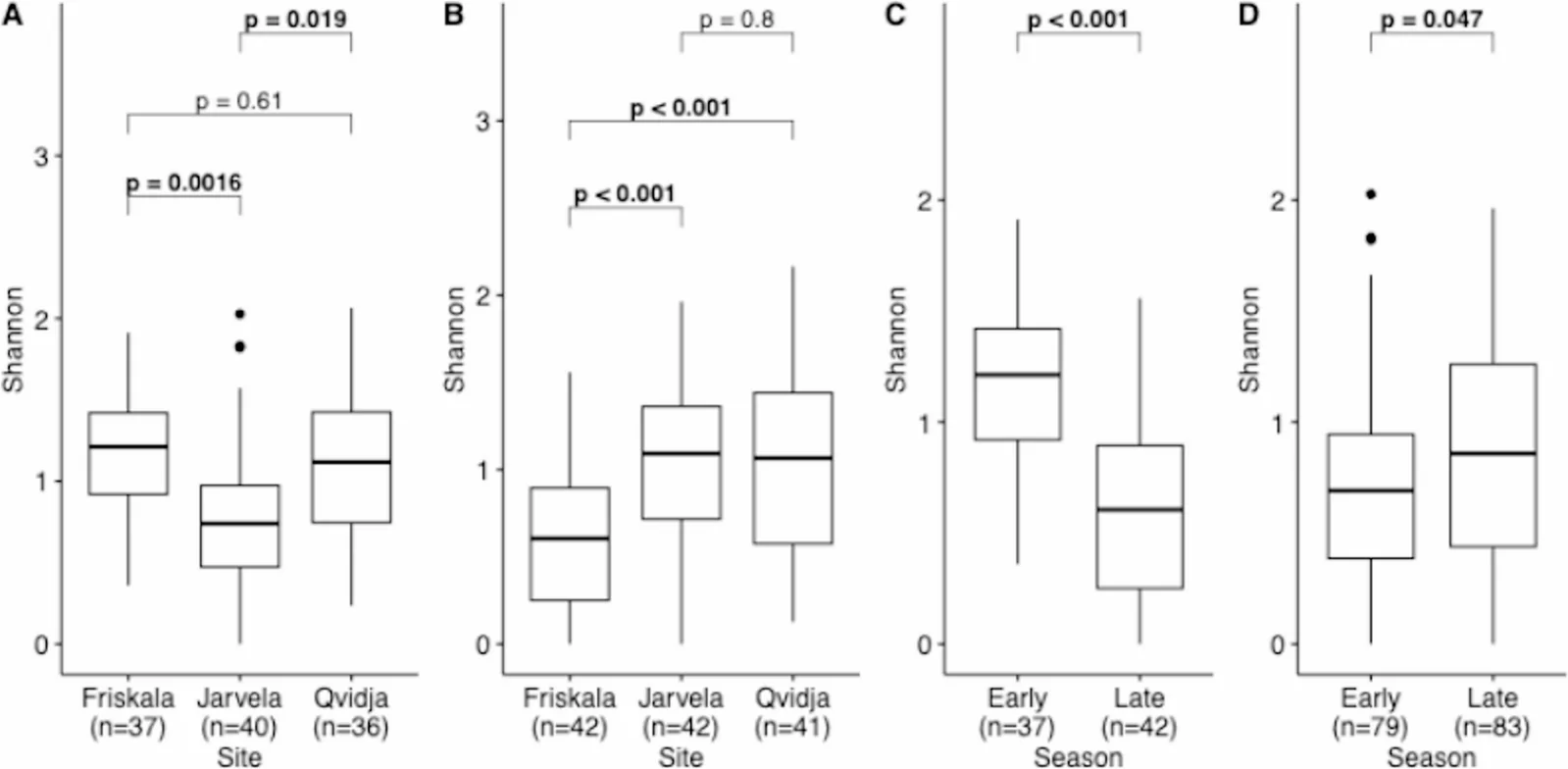
Comparison of prey Shannon diversity indices at genus-level for S. vulgatum across sites and seasons. Boxplots show (**a**) diversity between sites in early season, (**b**) diversity between sites in late season, seasonal comparison in (**c**) Friskala and (**d**) Järvelä. Statistically significant Holm-adjusted p-values are in bold

When comparing the sampling sites, odonates from Järvelä in the early season exhibited significantly lower diversity than those from Friskala or Qvidja (Fig. 4a). In the late season, however, odonates from Friskala had the lowest diversity (Fig. 4b). Furthermore, temporal differences were observed in odonates from Friskala where the prey diversity was significantly higher in the early season than in the late season, while the opposite was the case in Järvelä (Fig. 4c, d).

### Bacterial Alpha-diversity Responses between the Odonate Species, Sampling Sites and Sampling Seasons

**Fig. 5:**
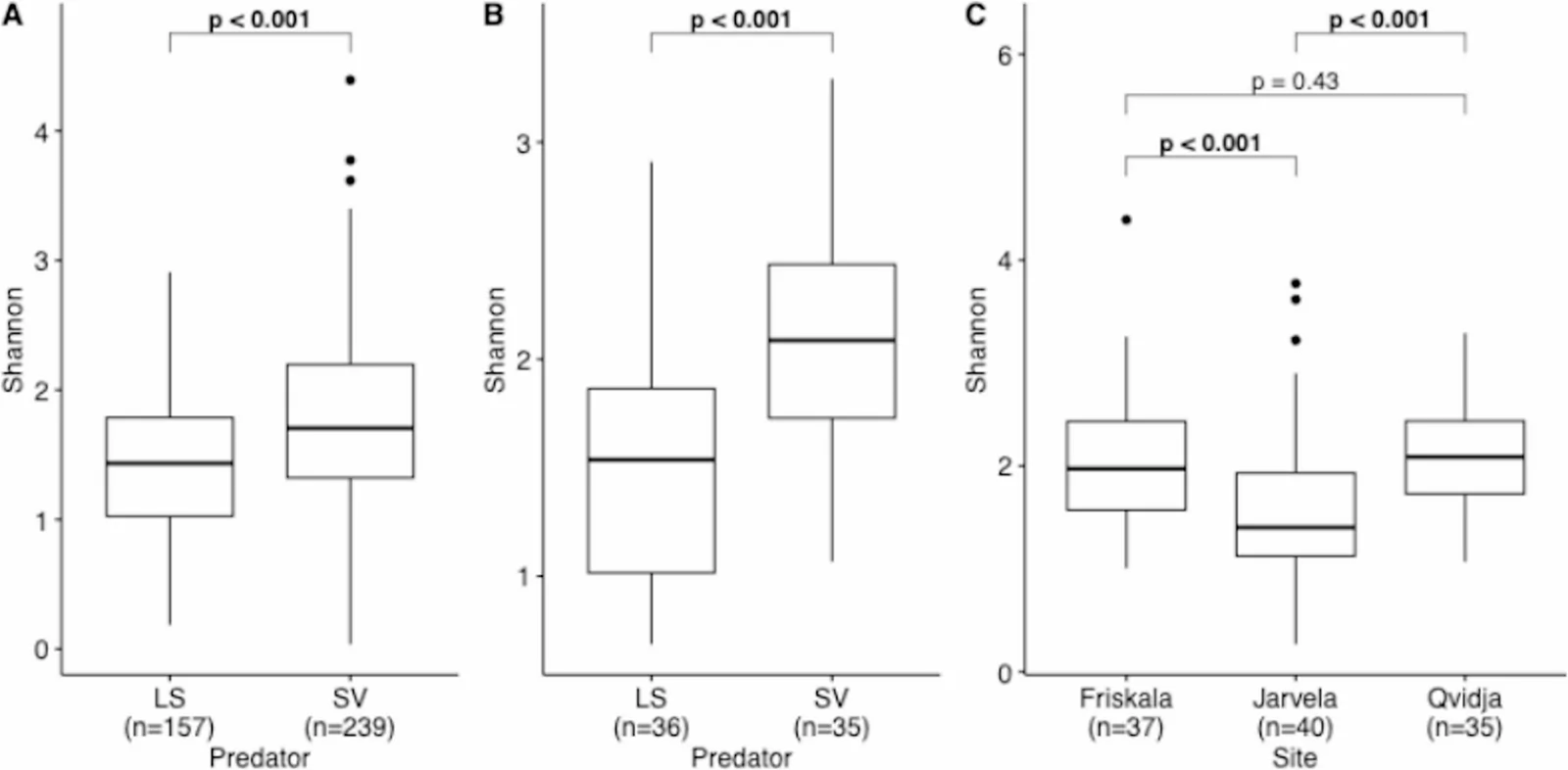
Comparison of bacterial Shannon diversity indices at ZOTU-level in L. sponsa and S. vulgatum. Boxplots show (**a**) diversity across all sampling sites and seasons, (**b**) diversity in early Qvidja, and (**c**) S. vulgatum diversity across different sampling sites in the early season. Statistically significant Holm-adjusted p-values are in bold

The bacterial Shannon diversity was altogether significantly higher in *S. vulgatum* than in *L. sponsa* (Fig. 5a), but across different sampling sites and seasons, the difference was statistically significant only in odonates from Qvidja in early season (Fig. 5b). When comparing the early season sampling sites, odonates from Järvelä had a significantly lower diversity than those from Friskala and Qvidja (Fig. 5c).

**Fig. 6:**
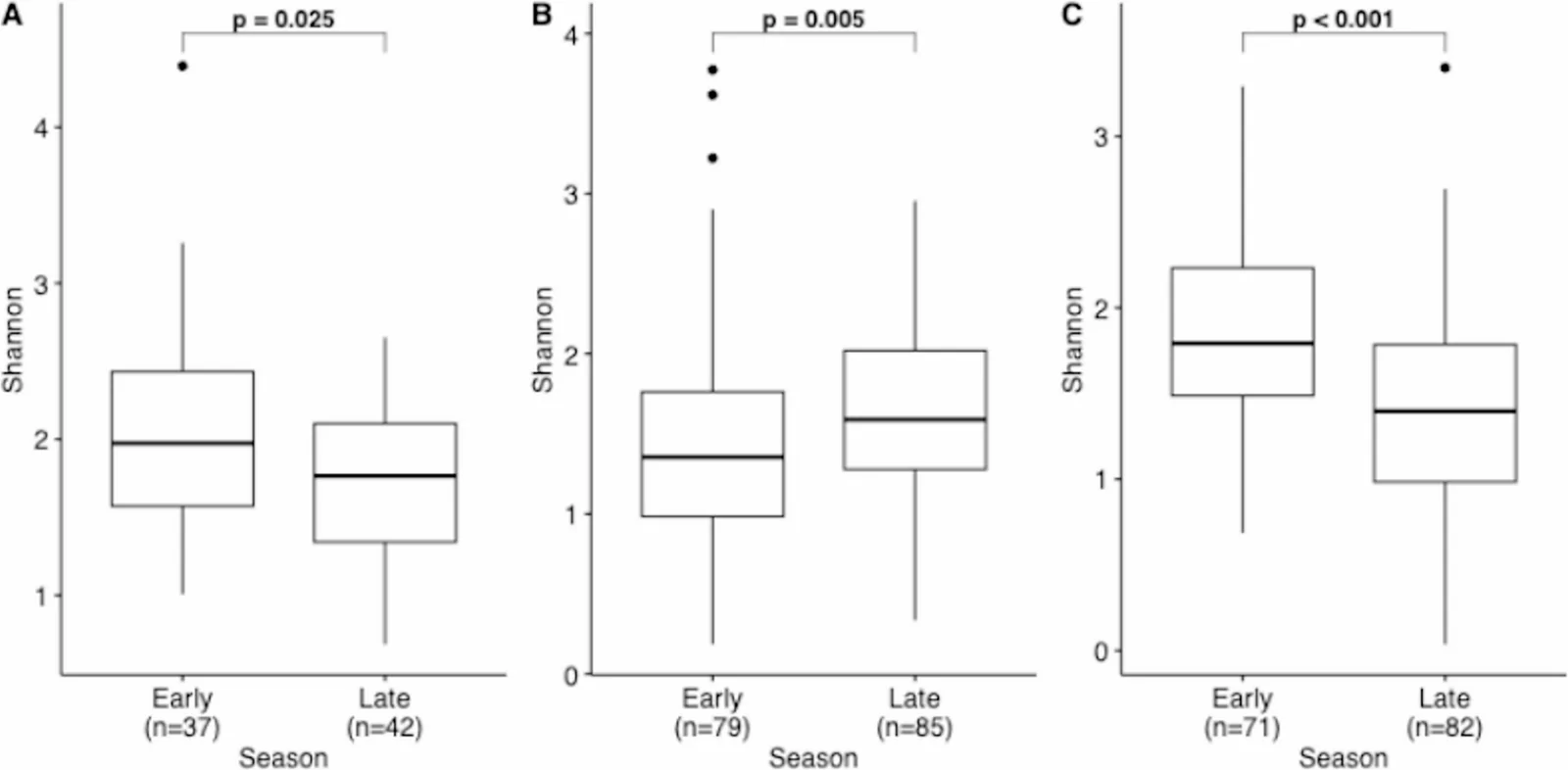
Comparison of bacterial Shannon diversity indices between early and late seasons. Boxplots show seasonal differences in (**a**) Friskala, (**b**) Järvelä, and (**c**) Qvidja. Statistically significant Holm-adjusted p-values are in bold

Comparing the bacterial Shannon diversities between the seasons showed that in Friskala and Qvidja the diversity was higher in the early season whereas in Järvelä the diversity was higher in the late season (Fig. 6).

### Correlation between Prey Diversity and Odonate Gut Microbiome Diversity

**Fig. 7:**
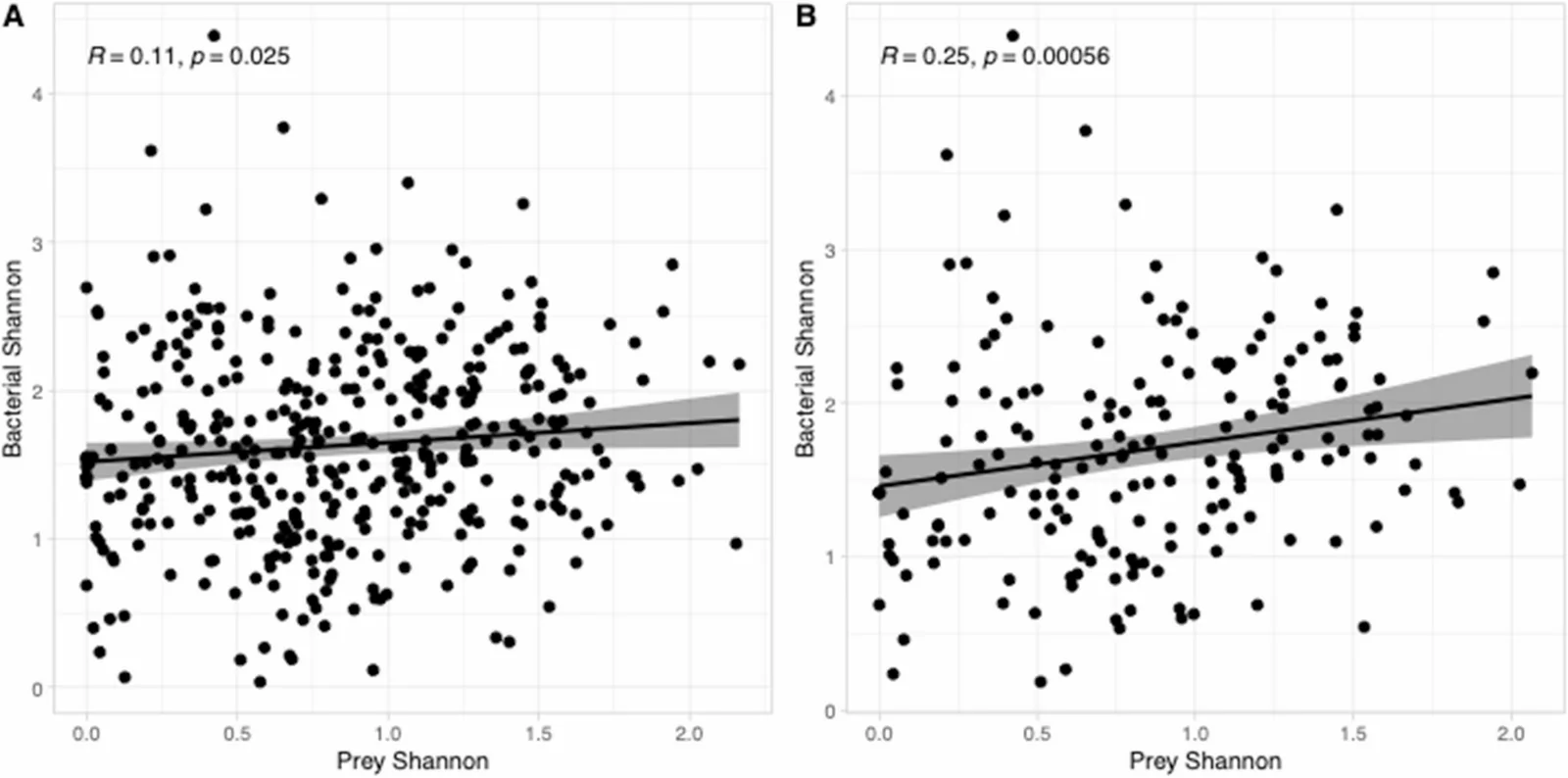
Correlation between the prey and bacterial Shannon diversity indices for Lestes sponsa and Sympetrum vulgatum in (**a**) all data, (**b**) early season. The 95% confidence interval is shown in grey

To study the relationship between the prey and bacterial Shannon diversities, Spearman rank correlation tests were conducted. Overall, there was a significant but weak correlation between the prey and bacterial diversities (S = 9043602, rho = 0.113, *p* = 0.025, Fig. 7a). The correlation was the strongest and most significant in the early season samples (S = 817360, rho = 0.250, *p* < 0.001, Fig. 7b).

In some comparisons, prey and bacterial Shannon diversities exhibited similar patterns. Specifically, *S. vulgatum* showed higher diversity than *L. sponsa* and individuals from Järvelä had the lowest diversity in the early season compared to both other sampling sites and the late season. Additionally, individuals from Friskala demonstrated higher diversity in the early season than in the late season.

### Effect of Environmental Factors on Prey and Bacterial Community

**Fig. 8:**
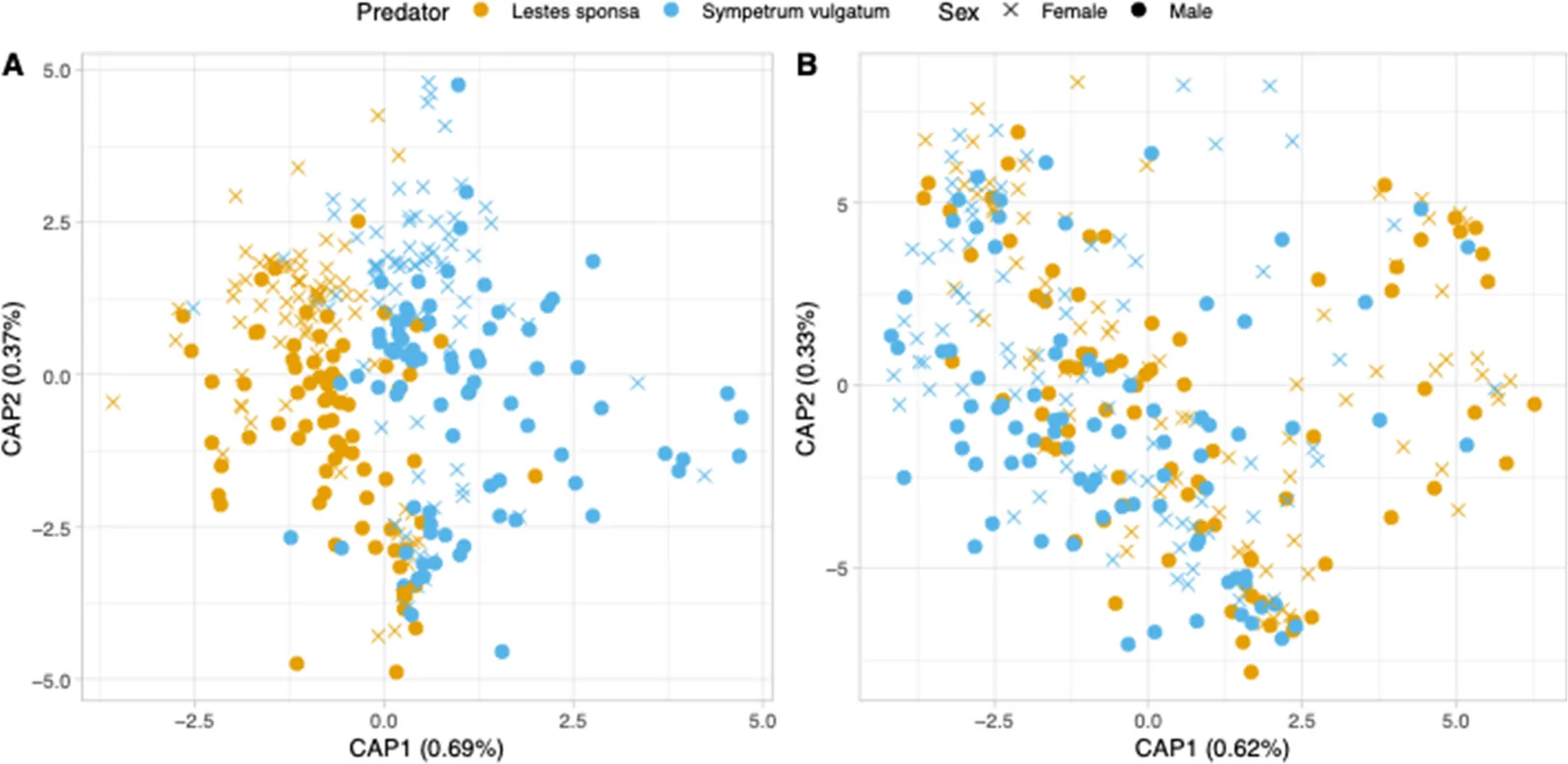
Constrained analysis of principal coordinates (CAP) ordination of the odonate (**a**) prey and (**b**) gut microbiome. Samples are indicated as coloured points with L. sponsa in yellow and S. vulgatum in blue. Female odonates indicated as crosses, male odonates as circles

The Constrained Analysis of Principal Coordinates (CAP) was used to assess the effect of environment – predator, season, location and sex – on the odonate diet. The environmental factors collectively explained 4% of the variation in the diet (ANOVA like permutation test for CAP F_5,389_ = 3.25, *p* = 0.001; adjusted R^2^ = 0.028). The prey species showing the strongest associations with the environmental variables were *Microchironomus tener*, *Phaonia tuguriorum*, *Procladius crassinervis*, *Coenosia pumila* and *Bryophaenocladius sp. 10ES*. Because season and site exhibited significant heterogeneity of dispersion, only predator species and sex were interpreted individually. Predator species had a significant effect on the odonate diet (ANOVA like permutation test for CAP F_1,313_=2.08, *p* = 0.001, Fig. 8a, Table S3).

We repeated the analysis for the bacteria and found that the environmental factors explained 3.2% of the variation in the gut microbiome (ANOVA like permutation test for CAP F_5,390_ = 2.62, *p* = 0.001; adjusted R^2^ = 0.020). The bacterial ZOTUs showing the strongest associations with the environmental variables belonged to *Klebsiella*, *Serratia*, *Enterobacteriaceae*, *Hafnia* and *Raoultella*. Again, as season and site exhibited significant heterogeneity of dispersion, only the significance of predator species and sex was assessed. Predator had a significant effect on the odonate gut microbiome (ANOVA like permutation test for CAP F_1,314_=1.96, *p* = 0.015; Fig. 8b, Table S4).

To portion out the potential effect of diet on the gut microbiota and assess the variation in the gut microbiota exclusively explained by the environmental factors, we performed partial CAP. The partial CAP model explained 2.3% of the variation in gut microbiome composition (ANOVA like permutation test for CAP F_5,364_ = 1.94, *p* = 0.001; adjusted R^2^ = 0.012).

### Community-level Responses between Prey and Bacterial Diversity

To evaluate the relationship between prey composition and gut microbiome composition, we conducted a Procrustes analysis on Lingoes-corrected Bray-Curtis ordinations. We detected a strong concordance between prey and gut microbiome beta diversity (PROTEST Procrustes correlation = 0.865, *p* = 0.001), indicating that individuals with more similar diets harboured more similar gut microbiomes. Congruence was slightly stronger in the early season (PROTEST Procrustes correlation = 0.850, *p* = 0.001) than in the late season (PROTEST Procrustes correlation = 0.810, *p* = 0.001), and Procrustes residuals confirmed a significant seasonal effect (ANOVA F_1,388_ = 4.03, *p* = 0.045).

**Fig. 9:**
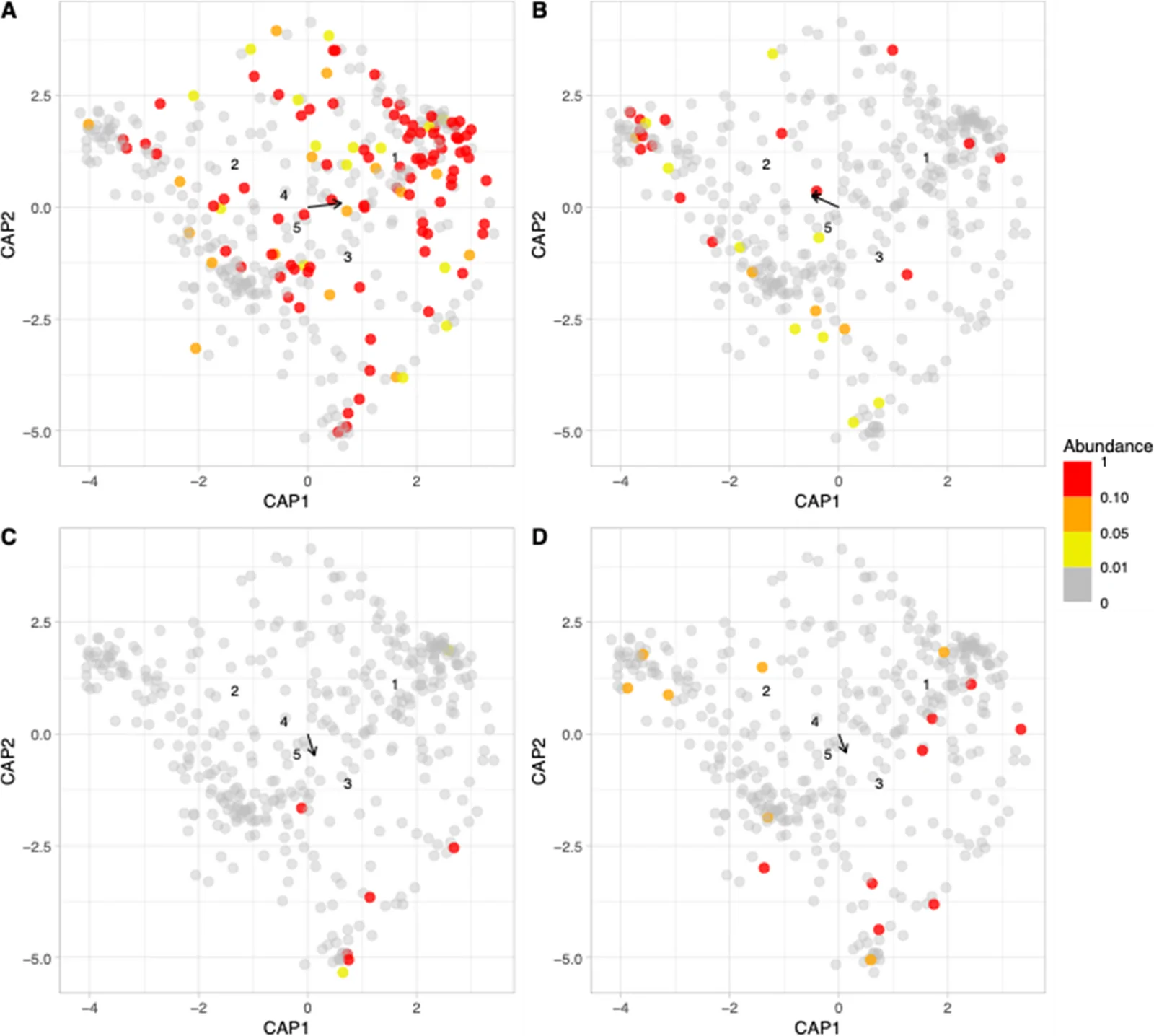
Constrained analysis of principal coordinates (CAP) ordinations of odonate gut microbiome. Dots represent individual samples, with the colour signifying the abundance of the four most influential prey genera: (**a**) Microchironomus, (**b**) Hybos, (**c**) Paratanytarsus and (**d**) Endochironomus. The arrow indicates the direction of the effect of the prey genus. The numbers indicate the bacterial ZOTUs showing the strongest associations with the diet and belong to: (1) Klebsiella, (2) Enterobacteriaceae (Zotu2), (3) Serratia, (4) Hafnia and (5) Enterobacteriaceae (Zotu 13)

To analyse patterns between the odonate prey and gut microbiome, we conducted constrained analysis of principal coordinates (CAP) with bacterial ZOTU-level Bray-Curtis dissimilarity indices as the response variable and a selection of prey abundances at the genus-level as the explanatory factors. The analysis revealed that the prey abundances explained 11% of the variation (ANOVA like permutation test for CAP F_24,369_=1.90, *p* = 0.001; adjusted R^2^ = 0.052) with various prey genera having a significant effect on the bacterial community compositions (Supplementary material, Table 5). Some of these genera were of very low abundance and/or rare, hence the analysis was repeated with subset of prey that were present in at least three samples and had at least one sample where the abundance was ≥ 0.01. In this case the diet explained 8.0% of the gut microbiome variation (ANOVA like permutation test for CAP F_16,377_=2.04, *p* = 0.001; adjusted R^2^ = 0.041). In both analyses, the prey genera with the largest and most significant effect on the bacterial diversity included *Hybos*,* Microchironomus*,* Paratanytarsus and Endochironomus* (Fig. 9).

The analysis was repeated separately for *S. vulgatum* and *L. sponsa*. The diet significantly explained 9.1% of the gut microbiome variation in *S. vulgatum* and 14.6% in *L. sponsa* (ANOVA like permutation test for CAP F_12,224_=1.86, *p* = 0.001; adjusted R^2^ = 0.042 and F_12,144_=2.06, *p* = 0.001; adjusted R^2^ = 0.075, respectively). The sets of significant prey genera mostly differed between the odonates (Supplementary material, Tables 4 and 5). The most important genera identified in the analysis of the whole data – *Microchironomus*,* Hybos*,* Paratanytarsus and Endochironomus* – were all also significant in *L. sponsa*, whereas in *S. vulgatum* only the first two were identified as significant. The bacterial ZOTUs showing the strongest associations with the diet were mostly the same as in the analysis of the whole data and were all within the family of *Enterobacteriaceae*, apart from one *Lactobacillales* ZOTU in the *S. vulgatum* analysis (Supplementary material, Table 6).

## Discussion

We found differences in gut microbiota and prey composition between the two sympatric odonate species, *Sympetrum vulgatum* and *Lestes sponsa*. Both diet and gut microbiota were influenced by season, location, and host species. In addition, dietary composition was associated with variation in gut microbiota diversity in both focal species.

### Prey Diversity and Composition

Our findings reveal distinct trophic strategies between *S. vulgatum* and *L. sponsa*, with *S. vulgatum* exhibiting a significantly broader dietary range. This pattern is consistent with niche partitioning commonly observed among sympatric odonates, where species diverge in prey-size preferences and microhabitat use to reduce interspecific competition [38, 39]. The larger body size and greater flight capability of *S. vulgatum* may have contributed to its ability to capture a wider range of prey, which may help explain its broader dietary niche. A broader dietary range may enhance ecological resilience by allowing odonates to switch among available prey when resources become scarce, although such generalism may come at the cost of reduced efficiency in exploiting any single prey type. At the same time, broader diets can also reflect opportunistic feeding rather than optimal resource use, meaning that dietary breadth does not necessarily translate into higher performance [40].

We also detected substantial temporal and spatial variation in dietary diversity, with the strongest pattern observed in *S. vulgatum*. Seasonal shifts in prey diversity at individuals from both Friskala and Järvelä likely reflect changes in prey availability driven by varying environmental conditions between sampling locations. Together, these patterns underscore the dynamic nature of odonate diets and highlight the influence of spatial and seasonal factors on prey communities.

Diptera dominated as the most common prey order across samples, aligning with prior research on odonate feeding ecology [18, 19]. Yet considerable variation occurred between biological replicates, predator species, and sites (Fig. 2a), indicating dietary flexibility and opportunistic feeding. This interpretation is supported by environmental factors explaining only ~ 4% of dietary variation, coupled with relatively weak associations between diet and environmental predictors in the indicator species analysis.

Within this broader pattern of flexible prey use, the high proportion of ants in the odonate diet, particularly in *S. vulgatum* in early Friskala, may be explained by the timing of our sampling. Sampling likely coincided with the period shortly after ant nuptial flights, when large numbers of winged ants would have been available as prey. This temporal overlap between ant reproductive events and odonate sampling could therefore account for the unexpectedly high predation rates of ants observed.

### Sex Differences in Diet

Although we did not detect statistically significant differences in diet between the sexes of the focal odonates, some prey taxa, were more frequently associated with one sex than the other, such as fly genus *Sepsis* with the *S. vulgatum* females in early Friskala (Fig. 2a). Sex-based differences in odonate diet and foraging can arise because males and females face distinct reproductive and spatial constraints. Males are often considered as time minimizers with shorter foraging periods, because intake of energy beyond a certain net energy threshold value does not increase their potential reproductive success, and females as energy maximizers with longer foraging periods, since their reproductive success is an increasing function of their net energy [41]. Also, for many odonate species the males tend to forage in more open areas, which may affect the available prey species [40]. Specifically, in *L. sponsa* it was shown that males are more abundant near the shoreline while females are more abundant in the areas further away from the shoreline [42]. Overall, the sex-specific differences in odonate diet are often driven by numerous rare prey taxa rather than by strong or consistent dietary segregation [43].

### Gut Microbiome Assemblages

Enterobacteriales, particularly Enterobacteriaceae, dominated across all sample types (Fig. 1b). This group includes many gut-associated bacteria found in insects and other animals [43, 44]. The prevalence of Lactobacillales and Pseudomonadales as secondary dominant orders aligns with findings from our previous studies on odonate gut microbiota [21]. Although we found some associations between specific bacterial taxa and environmental factors between the locations – the bacterial genus *Asaia* to be associated with Friskala and *Ferruginibacter* with early Friskala – overall only 3.2% of the variation in the gut microbiome could be explained by the environment. Some of this effect was likely caused by the prey, as once the effect of the diet was partialled out, environment explained only 2.3% of the variation in the gut microbiota. The ZOTUs most affected by the environment belonged to the family *Enterobacteriaceae*, the only family present in all samples.

### Gut Microbiome Diversity

Bacterial Shannon diversity was consistently higher in *S. vulgatum* than in *L. sponsa*, mirroring the pattern observed in prey diversity, although differences were not significant at individual sites or sampling seasons. Similar links between prey and bacterial alpha diversity have been reported in other studies comparing odonate species [45]. Notably, early-season sampling at Friskala yielded significantly higher bacterial and prey diversities than late-season sampling, while Järvelä showed the lowest diversity in the early season compared to other sites and the late season at Järvelä. These patterns suggest a potential connection between dietary diversity and gut bacterial richness, though the overall statistical association was weak. The correlation was strongest during the early sampling season, indicating that environmental drivers may partly mediate the diet–microbiota relationship. Such seasonal variation likely holds adaptive significance: as environmental conditions shift, predators may switch among prey sources to meet nutritional demands [46], with cascading effects on gut microbiota composition.

### Linking Diet and Gut Microbiota Variation

In other study organisms, spatiotemporal variation in diet and environment has likewise been shown to structure gut microbiota. In North American red squirrels, spatial location explained a large share of the variation in gut microbial composition, with environmental transmission of microbiota likely contributed to the observed spatiotemporal distribution in the gut community [38]. In mosquitoes, Maigoro et al. reported clear differences in gut microbial communities among regions and seasonal shifts in bacterial genera such as Asaia and Pseudomonas, which were correlated with host mosquito abundance and local environmental conditions [39]. Furthermore, in lab‑reared *Trichoplusia ni* moths, diet was shown to influence both the structure and putative function of gut microbial communities, with distinct diets producing different microbiota compositions in the host and indicating that some gut microbes are acquired directly from the diet [40].

In our study, the correlation between the diet and gut microbiome was not limited to alpha diversity, but we also saw a high Procrustes correlation between the two, suggesting a coupled pattern between the dietary composition and the gut microbiota composition. We also found various prey genera to have a significant effect on the bacterial communities. These genera explained approximately 11% of the variation in the gut microbiome, hence diet had a bigger impact than the environmental factors between the sampling locations. The bacterial taxa most affected by the prey belonged to the family *Enterobacteriaceae* and mostly were the same ZOTUs as the taxa most affected by the environment. When the analysis was performed separately for the two focal odonate species, the majority of the significantly expressed prey genera differed between them.

Two of the prey genera that significantly affected gut microbiome composition were also associated with environmental conditions in the indicator species analysis: *Microchironomus* with the early sampling season and *Anoecia* with *S. vulgatum*. This adds to the evidence that at least some of the variation in the gut microbiome caused by environment could be driven by a higher presence of certain prey species. Slightly different diets of the two odonate species or slightly different food availability driven by seasonal changes (or sampling site) could shift the gut microbiome.

### Ecological Drivers of Microbial Flexibility

The balance between stability and plasticity in gut microbiota is a critical area for discussion, particularly given that many insect-associated microbes are environmentally acquired and may vary in persistence and function (e.g [47]). Our results suggest that gut microbiota can exhibit both stability over time and plasticity in response to dietary changes across seasons. This adaptability may enhance host fitness by optimizing nutrient absorption from diverse diets. Moreover, environmental factors likely mediate the intricate relationships between diet and microbiota composition. For instance, variations in temperature, water quality, or habitat structure could influence prey availability and thus affect both dietary choices and microbial community dynamics at a broader ecological scale.

### Conclusion and Future Perspectives

Looking ahead, several promising research directions emerge. Longitudinal studies tracking diet–microbiota dynamics across diverse habitats and environmental conditions are needed to uncover the evolutionary patterns of odonate diet and gut microbial communities. Experimental manipulations and advanced genomic approaches, such as metagenomics, would allow for in-depth analysis of the functional implications of gut microbiota in relation to host diet, shedding light on microbiome stability and its effects on odonate fitness and population dynamics. Furthermore, interdisciplinary studies that integrate microbial ecology, dietary analysis, and environmental monitoring will be essential for developing a holistic understanding of insect host–microbiome interactions and their ecological significance.

Ultimately, our findings underscore the value of investigating spatial and temporal variation in gut microbiota and diet of insects, laying a strong foundation for future research with important applications in conservation, management, and the preservation of aquatic biodiversity.

## Supplementary Information

Below is the link to the electronic supplementary material.

Supplementary Material 1

## Data Availability

Labelled raw FASTQ reads, ZOTUs, ZOTU assignations, and ZOTU tables are available in the Dryad Digital Repository under the title: The Invisible Web: Linking Diet and Gut Microbiota Across Time and Space in Odonates, and under https://doi.org/10.5061/dryad.wpzgmsc21.
