# Supplementary material for: The Invisible Web: Linking Diet and Gut Microbiota Across Time and Space in Odonates
Source: Microb Ecol. 2026 Aug 4;89:203. doi: 10.1007/s00248-026-02850-y (PMC13743688; doi:10.1007/s00248-026-02850-y)
Supplement: Supplementary file 1 [file 248_2026_2850_MOESM1_ESM.docx]

Supplementary materials for:

**The Invisible Web: Linking Diet and Gut Microbiota Across Time and Space in Odonates**

*Sample Processing and Molecular Analyses*

To characterize the bacterial assemblages and dietary composition of the focal species, we used established metabarcoding protocols for dragonflies and damselflies building on earlier optimizations [1, 2]. Even though odonate feces are tiny, we did not pool individual samples but processed all samples from each individual separately to gain information on individual variation. For DNA extraction, we used the NucleoSpin Tissue XS Kit (product nr 740901, Macherey-Nagel, Düren, Germany).

Each DNA sample was amplified in two separate reactions that were individually tagged and sequenced for each primer pair (altogether six reactions per sample). The first round of COI-targeting PCR was conducted according to Kankaanpää et al. [28], and bacterial 16S rRNA gene amplification followed Morrill et al. [24]. In the second PCR round, the first PCR products were modified by attaching Illumina-specific adapters and sample-specific indices. Then, all the indexed reactions were first pooled per primer pair and replicate and then purified using magnetic beads [29].

We used two primer pairs targeted to the prey mitochondrial cytochrome c oxidase subunit I (COI) gene. Two primer pairs were chosen to achieve more comprehensive taxonomic coverage and to add robustness to the dietary results. The first primer pair was ANML pair LCO1490 [3] and CO1-CFMRa [4], and targeted the first half of the COI barcode region. The second primer pair was Leray pair mlCOIintF [5] and jgHCO2198 [6], and amplified the latter part of the COI barcode region. To characterize the microbiota, we amplified the hypervariable v4 region of the bacterial 16S rRNA gene, using primers 515F-Parada (also known as 515FB: 5′-GTG YCA GCM GCC GCG GTA A-3′; Parada et al. 2016) and 806R-Apprill (also known as 806RB: 5′-GGA CTA CNV GGG TWT CTA AT-3′ [7]. Each DNA sample was amplified in two separate reactions that were individually tagged and sequenced for each primer pair (altogether six reactions per sample). The setup of the first round of PCR followed the protocols from Kankaanpää et al. [8] and included 5 μl of 2× MyTaq HS Red Mix (Bioline, UK), 2.4 μl of H2O, 150 nM of each primer (two forward and two reverse primer versions; total primer mix concentration 600 nM), and 2 μl of DNA extract per each sample in 10 μl volume. Primer-specific cycling conditions were for ANML: 60 sec at 95°C, then 5 cycles at 95°C for 60 sec, 45°C for 90 sec and 72 °C for 90 sec, followed by 35 cycles at 95°C for 60 sec, 50°C for 90 sec and 72°C for 60 sec, followed by 72°C for 10 min. For Leray: 5 min at 95°C, then 16 cycles at 95°C for 10 sec, 61°C (decreased by 1°C per cycle) for 60 sec and 72°C for 30 sec, followed by 20 cycles at 46°C for 60 sec and 72°C for 30 sec, followed by 72°C for 10 min. For bacterial 16S primers 515FB+806RB: 3 min at 95°C, then 35 cycles of 45 s at 95°C, 1 min at 50°C, and 1 min 30 s at 72°C, ending with 10 min at 72 °C [following methods from our previous publication 2].

In the second PCR round, the first PCR products were modified by attaching Illumina-specific adapters and sample-specific indices. For a reaction volume of 10 μl in the indexing PCR, we mixed 5 μl of MyTaq HS RedMix, 500 nM of each tagged and indexed primer (i7 and i5), and 3 μl of locus-specific PCR product from the first PCR phase. For this second PCR round, we used the following protocol: initial denaturation for 3 min at 98 °C, then 15 cycles of 20 s at 95 °C, 15 s at 60 °C, and 30 s at 72 °C, followed by 3 min at 72 °C. Then, all the indexed reactions were first pooled per primer pair and replicate and then purified using magnetic beads [9, 10]. Sequencing was done on two Illumina MiSeq v3 PE 2×300 bp runs (Illumina Inc., San Diego, CA, USA), including the PhiX control library by the Finnish Functional Genomics Centre, Turku Bioscience, University of Turku and Åbo Akademi University, Turku, Finland.

*Read processing*

After sequencing, the reads were demultiplexed into each original sample and uploaded onto CSC servers (IT Center for Science, [www.csc.fi](http://www.csc.fi)) for bioinformatic analysis. Paired-end reads (for each primers: ANML: 5,259,261; Leray: 11,028,398; Bac16S: 9,332,092) were merged and trimmed for quality using 64-bit VSEARCH version 2.14.2 [11] command ‘*fastq_mergepairs*’ with the default options and ‘*fastq_allowmergestagger*’, and truncate length of 220 bp for Bac16S reads. Before merging step, ANML and Leray reads were combined into the same batch and processed together. Primers were removed from the merged reads using software cutadapt version 1.14 [12] with 20% mismatch rate and minimum length after trimming 100 bp. Trimmed reads were then collapsed into unique sequences (singletons removed) with command ‘fastx_uniques’ and option ‘minuniquesize’ set to 2. Finally, reads were corrected for point errors to obtain an accurate set of amplicon sequences (=denoised) and filtered of chimeric amplicons (=chimeras were removed) resulting in sequence variants called ZOTUs (‘ZOTU’, ‘zero-radius OTU’) through command ‘unoise3’ using USEARCH version 11 with settings minsize = 8 and unoise_alpha = 2.

The ZOTUs were mapped back to the original trimmed reads with command ‘usearch_global’ to establish the total number of reads in each sample using VSEARCH. We were able to map 5,024,908 of 5,137,746 (97.80%) of ANML reads, 7,817,794 of 7,875,296 (99.27%) of Leray reads, and 8,771,659 of 9,153,656 (95.83%) of Bac16S reads to our original samples. The ZOTUs (sequence variants) were assigned to taxa using different database depending on the locus. Both ANML and Leray ZOTUs (mtDNA COI gene) were identified using BOLD Systems database with a custom API script [13, 14]. The similarity thresholds for assigned identification varied according to taxonomic level: species 98%, genus 96%, family 90%, order 85%, class 80%, and phylum 70%. The database ‘16S RDP training set v18’ (21k seqs) was downloaded from the USEARCH website (drive5.com/usearch/manual/sintax_downloads.html) and used for assignation of Bac16S ZOTUs (ribosomal sRNA gene) with SINTAX probabilistic algorithm implemented in VSEARCH [15]. For the chosen 16S database, the genus level is the lowest taxonomic level. The probability thresholds for assigned identification varied according to taxonomic level: species 90%, genus 85%, family 80%, order, class, phylum, and domain 80%. The data was further filtered to remove artefacts, spurious reads, and nontargets based on information on the numerous control samples, technical replicates, and taxonomy. First, we removed those ZOTUs from any sample that had fewer reads than extraction or PCR controls. Then, we collapsed reads based on the taxonomy per each sample, that is, all the reads that were assigned to the same taxa per sample were summarized. Then, we removed taxa that were present in a sample by only one of the two replicates and finally summed the reads in both replicates. Then, to remove potentially leaked ‘tag-jumped’ (sample cross-talk) reads from the data, we removed all taxa from the samples with less than 0.05% proportion of the total reads in one sample. We removed all the taxa outside Phylum Arthropoda (COI reads: primer pairs ANML and Leray) or domains Bacteria or Archaea, as well as Class Chloroplast (Bac 16S reads: primer pair 515+806). Finally, very rare occurrences (sequence count < 2) were removed (ANML+Leray: 5,156,086 final reads; Bac16S: 8,316,871 final reads). Labelled raw FASTQ reads, ZOTUs, ZOTU assignations, and ZOTU tables are available in the Dryad Digital Repository and SRA in NCBI.

*Statistical analyses*

The statistical analyses of the data were performed using R v. 4.2.3 and Rstudio [16, 17] on total sum scaled data. Dufrene-Legendre Indicator Species Analysis for both the prey and the bacterial data was performed using R package *labdsv* v.2.1-0 [18]. For the analyses, relative abundance data at the genus-level (where possible) were used. The p-values were adjusted using the Holm correction. Significant indicators were determined as p ≤ 0.05 and indicator value ≥ 0.3. The alpha diversity analysis for both prey (at genus-level) and bacteria (at ZOTU-level) was performed using R package *vegan* v.2.6-4 [19] and the significance of the differences between focal odonates, sites and sampling seasons were analyzed using packages *ggpubr* v.0.6.0 [20] and *rstatix* v.0.7.2 [21] with two-sided Wilcoxon rank-sum test with multiple comparison correction. Where the Wilcoxon rank-sum test was significant, the package *car* v.3.1-2 [22] was used for the Levene's Test, which showed there were no significant differences between the group variances. To test for a correlation between dietary and gut bacterial Shannon diversity we performed Spearman rank correlation test for all the data as well as data subset by season, site and predator.

The constrained analysis of principal coordinates (CAP) was performed using the package *vegan* v.2.6-4 method *capscale()* on Bray-Curtis dissimilarity indices at species-level (where possible) for the prey and ZOTU-level for the bacteria. For the prey, if no species information was available, ZOTU was used. Prior to the CAP, we assessed the multivariate homogeneity of group dispersions using the *vegan* methods *betadisper()* and *permutest()*. The *betadisper* results were significant for both the sampling season and site for both prey and bacteria, hence only predator and sex were used as the explanatory factors in the CAP. As the sampling site Friskala had no *L. sponsa*, the samples collected in Friskala were not included in the analysis. The significance of the results was tested using ANOVA-like permutation test with 999 permutations. To assess the overall effect of the environment, CAP was performed with all the environmental explanatory factors, but the significance of individual terms was not assessed. To partial out the potential effect of the prey and to assess the level of variation in the gut microbiota exclusively explained by the environmental factors, partial CAP was performed, by adding a conditioning matrix with a selection of prey genera abundances to the *capscale()* formula. The prey genera was chosen using the package *vegan* method *ordistep()* (as explained in the next paragraph).

CAP was also used to test if the prey had a significant effect on gut bacterial community compositions and to identify which prey genera had a significant effect on the gut microbiome. Here we used bacterial Bray-Curtis dissimilarity indices at ZOTU-level as the response variable and a selection of prey abundances at the genus-level as the explanatory factors. The prey was selected and model for the CAP was built using the package *vegan* method *ordistep().* The analysis was repeated with the subset of prey that were present in at least three samples and had at least one sample where the abundance was ≥ 0.01. Separate models were also built for both of the odonates. The significance of the results was tested using ANOVA-like permutation test with 999 permutations.

The relationship between prey composition and gut microbiome composition was also evaluated using Procrustes analysis from the package *vegan*. Bray–Curtis dissimilarity matrices were calculated independently for prey (genus-level) and bacterial communities (ZOTU-level) and ordinated using weighted principal coordinates analysis (method *wcmdscale()*). To account for the non-Euclidean properties of Bray–Curtis dissimilarities, a Lingoes correction was applied. The resulting ordination configurations were compared using Procrustes rotation, and statistical significance was assessed using the permutation-based method *protest()* with 999 permutations. Analyses were performed for the full dataset and repeated separately for seasons and predator species. Differences in prey–microbiome congruence among different environmental factors were evaluated by analysing Procrustes residuals using ANOVA with predator species, sex, season and site as explanatory factors.

Table S1. Odonate prey Dufrene-Legendre indicator species analysis indicator values (indval) for different sample types. Comparison significances indicated as raw p values and Holm adjusted p values. Only the genera that were identified as an indicator species are included. Indval ranges from 0 (bad indicator) to 1 (good indicator).

| Comparing | Genus | Sample type | Indval | p value | p holm |
| --- | --- | --- | --- | --- | --- |
| Predator | Anoecia | *Lestes sponsa* | 0.002 | 0.0001 | 0.046 |
|  | Anoecia | *Sympetrum vulgatum* | 0.379 |  |  |
| Season | Microchironomus | Early | 0.392 | 0.0001 | 0.046 |
|  | Microchironomus | Late | 0.039 |  |  |
| Predator + Site + Season | Bryophaenocladius | *Lestes sponsa* Jarvela Early | 0 | 0.0001 | 0.046 |
|  | Bryophaenocladius | *Lestes sponsa* Jarvela Late | 0 |  |  |
|  | Bryophaenocladius | *Lestes sponsa* Qvidja Early | 0.046 |  |  |
|  | Bryophaenocladius | *Lestes sponsa* Qvidja Late | 0 |  |  |
|  | Bryophaenocladius | *Sympetrum vulgatum* Friskala Early | 0.013 |  |  |
|  | Bryophaenocladius | *Sympetrum vulgatum* Friskala Late | 0 |  |  |
|  | Bryophaenocladius | *Sympetrum vulgatum* Jarvela Early | 0 |  |  |
|  | Bryophaenocladius | *Sympetrum vulgatum* Jarvela Late | 0.004 |  |  |
|  | Bryophaenocladius | *Sympetrum vulgatum* Qvidja Early | 0.398 |  |  |
|  | Bryophaenocladius | *Sympetrum vulgatum* Qvidja Late | 0 |  |  |
|  | Lasius | *Lestes sponsa* Jarvela Early | 0 | 0.0001 | 0.046 |
|  | Lasius | *Lestes sponsa* Jarvela Late | 0.001 |  |  |
|  | Lasius | *Lestes sponsa* Qvidja Early | 0.002 |  |  |
|  | Lasius | *Lestes sponsa* Qvidja Late | 0 |  |  |
|  | Lasius | *Sympetrum vulgatum* Friskala Early | 0.313 |  |  |
|  | Lasius | *Sympetrum vulgatum* Friskala Late | 0 |  |  |
|  | Lasius | *Sympetrum vulgatum* Jarvela Early | 0 |  |  |
|  | Lasius | *Sympetrum vulgatum* Jarvela Late | 0 |  |  |
|  | Lasius | *Sympetrum vulgatum* Qvidja Early | 0.008 |  |  |
|  | Lasius | *Sympetrum vulgatum* Qvidja Late | 0 |  |  |
| Predator + Site + Season + Sex | Sepsis | *Lestes sponsa* Jarvela Early Female | 0 | 0.0001 | 0.046 |
|  | Sepsis | *Lestes sponsa* Jarvela Early Male | 0 |  |  |
|  | Sepsis | *Lestes sponsa* Jarvela Late Female | 0 |  |  |
|  | Sepsis | *Lestes sponsa* Jarvela Late Male | 0 |  |  |
|  | Sepsis | *Lestes sponsa* Qvidja Early Female | 0 |  |  |
|  | Sepsis | *Lestes sponsa* Qvidja Early Male | 0 |  |  |
|  | Sepsis | *Lestes sponsa* Qvidja Late Female | 0 |  |  |
|  | Sepsis | *Lestes sponsa* Qvidja Late Male | 0 |  |  |
|  | Sepsis | *Sympetrum vulgatum* Friskala Early Female | 0.3 |  |  |
|  | Sepsis | *Sympetrum vulgatum* Friskala Early Male | 0 |  |  |
|  | Sepsis | *Sympetrum vulgatum* Friskala Late Female | 0 |  |  |
|  | Sepsis | *Sympetrum vulgatum* Friskala Late Male | 0 |  |  |
|  | Sepsis | *Sympetrum vulgatum* Jarvela Early Female | 0 |  |  |
|  | Sepsis | *Sympetrum vulgatum* Jarvela Early Male | 0.002 |  |  |
|  | Sepsis | *Sympetrum vulgatum* Jarvela Late Female | 0 |  |  |
|  | Sepsis | *Sympetrum vulgatum* Jarvela Late Male | 0.003 |  |  |
|  | Sepsis | *Sympetrum vulgatum* Qvidja Early Female | 0 |  |  |
|  | Sepsis | *Sympetrum vulgatum* Qvidja Early Male | 0 |  |  |
|  | Sepsis | *Sympetrum vulgatum* Qvidja Late Female | 0.004 |  |  |
|  | Sepsis | *Sympetrum vulgatum* Qvidja Late Male | 0 |  |  |

Table S2. Odonate gut microbiome Dufrene-Legendre indicator species analysis indicator values (indval) for different sample types. Comparison significances indicated as raw p values and Holm adjusted p values. Only the genera that were identified as an indicator species are included. Indval ranges from 0 (bad indicator) to 1 (good indicator).

| Comparing | Genus | Sampletype | Indval | p.value | p.holm |
| --- | --- | --- | --- | --- | --- |
| Site | Asaia | Friskala | 0.405 | 0.0001 | 0.028 |
|  | Asaia | Jarvela | 0.047 |  |  |
|  | Asaia | Qvidja | 0.091 |  |  |
| Predator + Season + Site | Ferruginibacter | *Lestes sponsa* Early Jarvela | 0 | 0.0001 | 0.028 |
|  | Ferruginibacter | *Lestes sponsa* Early Qvidja | 0 |  |  |
|  | Ferruginibacter | *Lestes sponsa* Late Jarvela | 0 |  |  |
|  | Ferruginibacter | *Lestes sponsa* Late Qvidja | 0 |  |  |
|  | Ferruginibacter | *Sympetrum vulgatum* Early Friskala | 0.371 |  |  |
|  | Ferruginibacter | *Sympetrum vulgatum* Early Jarvela | 0 |  |  |
|  | Ferruginibacter | *Sympetrum vulgatum* Early Qvidja | 0 |  |  |
|  | Ferruginibacter | *Sympetrum vulgatum* Late Friskala | 0 |  |  |
|  | Ferruginibacter | *Sympetrum vulgatum* Late Jarvela | 0 |  |  |
|  | Ferruginibacter | *Sympetrum vulgatum* Late Qvidja | 0 |  |  |

Table S3. The five prey taxa showing the strongest associations with predator species and sex in the constrained ordination. The table includes the coordinates of each taxon on the first two constrained axes (CAP1 and CAP2) and its distance from the ordination origin, calculated from these coordinates.

| Taxon | CAP1 | CAP2 | Distance |
| --- | --- | --- | --- |
| Coenosia pumila | -0.380456248 | 0.009747295 | 0.3805811 |
| Microchironomus tener | 0.009751847 | -0.333071744 | 0.3332145 |
| Anoecia_Zotu37 | 0.319951703 | 0.034703326 | 0.3218282 |
| Anoecia_Zotu21 | 0.282598724 | -0.127053945 | 0.3098463 |
| Scaptomyza sp. Dip_32 AV-2017 | 0.140897517 | 0.271637629 | 0.3060051 |

Table S4. The five bacterial ZOTUs showing the strongest associations with predator species and sex in the constrained ordination. The table includes the coordinates of each ZOTU on the first two constrained axes (CAP1 and CAP2) and its distance from the ordination origin, calculated from these coordinates.

| ZOTU | CAP1 | CAP2 | Distance |
| --- | --- | --- | --- |
| Serratia_Zotu3 | 0.8902930 | 0.2799692 | 0.9332762 |
| Klebsiella_Zotu1 | 0.5592141 | -0.4793358 | 0.7365346 |
| Hafnia_Zotu4 | 0.1258283 | 0.3276723 | 0.3510013 |
| Enterobacteriaceae_Zotu2 | -0.1720847 | 0.2659377 | 0.3167586 |
| Lactobacillales_Zotu6 | -0.1025679 | -0.2525372 | 0.2725715 |

Table S5. ANOVA-like permutation test results for the constrained analysis of principal coordinates of the odonate prey genera with a significant effect on the gut microbiome. The test was applied to genera, but family and order are given as reference for each genus. The table also includes the number of samples each genus was present in (n) as well as their abundance range in those samples (abundance).

| Order | Family | Genus | Df | SumOfSqs | F | p | n | Abundance |
| --- | --- | --- | --- | --- | --- | --- | --- | --- |
| Diptera | Chironomidae | Microchironomus | 1 | 1.616 | 4.036 | 0.001 | 131 | 0.001-1 |
| Diptera | Hybotidae | Hybos | 1 | 1.310 | 3.272 | 0.001 | 35 | 0.001-0.999 |
| Diptera | Chironomidae | Paratanytarsus | 1 | 0.955 | 2.385 | 0.001 | 18 | 0.001-0.708 |
| Diptera | Chironomidae | Chaetocladius | 1 | 0.653 | 1.630 | 0.001 | 1 | 0.011 |
| Diptera | Agromyzidae | Agromyza | 1 | 0.757 | 1.890 | 0.001 | 3 | 0.009-0.236 |
| Hemiptera | Miridae | Apolygus | 1 | 0.631 | 1.577 | 0.002 | 1 | 0.168 |
| Coleoptera | Curculionidae | Rhamphus | 1 | 0.640 | 1.599 | 0.002 | 1 | 0.115 |
| Diptera | Drosophilidae | Scaptomyza | 1 | 0.988 | 2.467 | 0.004 | 14 | 0.014-0.915 |
| Diptera | Chironomidae | Endochironomus | 1 | 0.873 | 2.180 | 0.004 | 19 | 0.001-0.995 |
| Araneae | Linyphiidae | Agyneta | 1 | 0.668 | 1.668 | 0.004 | 3 | 0.023-0.717 |
| Hymenoptera | Signiphoridae | Chartocerus | 1 | 0.631 | 1.575 | 0.005 | 1 | 0.059 |
| Hymenoptera | Figitidae | Alloxysta | 1 | 0.620 | 1.549 | 0.006 | 1 | 0.01 |
| Coleoptera | Chrysomelidae | Chaetocnema | 1 | 0.642 | 1.605 | 0.01 | 3 | 0.025-0.434 |
| Diptera | Sciaridae | Xylosciara | 1 | 0.626 | 1.563 | 0.011 | 3 | 0.004-0.045 |
| Diptera | Polleniidae | Pollenia | 1 | 0.780 | 1.947 | 0.012 | 11 | 0.003-0.982 |
| Hemiptera | Aphididae | Anoecia | 1 | 0.751 | 1.875 | 0.016 | 102 | 0.001-0.986 |
| Diptera | Lauxaniidae | Meiosimyza | 1 | 0.666 | 1.664 | 0.018 | 3 | 0.01-0.036 |
| Diptera | Chironomidae | Cryptochironomus | 1 | 0.674 | 1.682 | 0.022 | 10 | 0.003-0.701 |
| Araneae | Tetragnathidae | Tetragnatha | 1 | 0.711 | 1.776 | 0.026 | 17 | 0.001-0.947 |
| Hemiptera | Cicadellidae | Macrosteles | 1 | 0.629 | 1.570 | 0.034 | 2 | 0.041-0.107 |
| Diptera | Cecidomyiidae | Monardia | 1 | 0.602 | 1.503 | 0.035 | 10 | 0.001-0.216 |
| Hemiptera | Cicadellidae | Grypotes | 1 | 0.649 | 1.622 | 0.039 | 5 | 0.001-0.163 |
| Diptera | Pipunculidae | Pipunculus | 1 | 0.625 | 1.562 | 0.043 | 2 | 0.915-0.993 |
| Diptera | Chironomidae | Gymnometriocnemus | 1 | 0.584 | 1.459 | 0.049 | 2 | 0.039-0.116 |
|  |  | Residual | 369 | 147.733 |  |  |  |  |

Table S4. ANOVA-like permutation test results for the constrained analysis of principal coordinates of the Sympetrum vulgatum prey genera with a significant effect on the gut microbiome. The test was applied to genera, but family and order are given as reference for each genus. The table also includes the number of samples each genus was present in (n) as well as the abundance range in those samples (abundance).

| Order | Family | Genus | Df | SumOfSqs | F | p | n | Abundance |
| --- | --- | --- | --- | --- | --- | --- | --- | --- |
| Diptera | Chironomidae | Microchironomus | 1 | 1.469 | 3.711 | 0.001 | 80 | 0.001-1 |
| Sarcoptiformes | Chamobatidae | Chamobates | 1 | 0.613 | 1.548 | 0.002 | 1 | 0.033 |
| Hemiptera | Miridae | Apolygus | 1 | 0.614 | 1.550 | 0.003 | 1 | 0.168 |
| Diptera | Drosophilidae | Scaptomyza | 1 | 0.924 | 2.334 | 0.004 | 11 | 0.014-0.915 |
| Diptera | Sciaridae | Cratyna | 1 | 0.701 | 1.771 | 0.007 | 3 | 0.003-0.176 |
| Araneae | Linyphiidae | Agyneta | 1 | 0.630 | 1.591 | 0.007 | 3 | 0.023-0.717 |
| Araneae | Tetragnathidae | Tetragnatha | 1 | 0.713 | 1.801 | 0.015 | 4 | 0.019-0.26 |
| Coleoptera | Chrysomelidae | Chaetocnema | 1 | 0.603 | 1.522 | 0.019 | 3 | 0.025-0.434 |
| Diptera | Polleniidae | Pollenia | 1 | 0.678 | 1.712 | 0.021 | 10 | 0.003-0.982 |
| Diptera | Hybotidae | Hybos | 1 | 0.672 | 1.696 | 0.03 | 20 | 0.001-0.954 |
| Diptera | Chironomidae | Cryptochironomus | 1 | 0.613 | 1.547 | 0.035 | 5 | 0.003-0.701 |
| Hemiptera | Aphididae | Anoecia | 1 | 0.622 | 1.572 | 0.044 | 96 | 0.001-0.986 |
|  |  | Residual | 224 | 88.700 |  |  |  |  |

Table S5. ANOVA-like permutation test results for the constrained analysis of principal coordinates of the Lestes sponsa prey genera with a significant effect on the gut microbiome. The test was applied to genera, but family and order are given as reference for each genus. The table also includes the number of samples each genus was present in (n) as well as the abundance range in those samples (abundance).

| Order | Family | Genus | Df | SumOfSqs | F | p | n | Abundance |
| --- | --- | --- | --- | --- | --- | --- | --- | --- |
| Diptera | Agromyzidae | Agromyza | 1 | 0.717 | 1.998 | 0.001 | 2 | 0.074-0.236 |
| Lepidoptera | Gelechiidae | Palumbina | 1 | 0.655 | 1.826 | 0.001 | 1 | 0.003 |
| Diptera | Chironomidae | Paratanytarsus | 1 | 0.811 | 2.260 | 0.001 | 12 | 0.001-0.708 |
| Diptera | Chironomidae | Tanypus | 1 | 0.640 | 1.784 | 0.001 | 2 | 0.005-0.072 |
| Diptera | Phoridae | Borophaga | 1 | 0.651 | 1.814 | 0.001 | 1 | 0.94 |
| Diptera | Hybotidae | Hybos | 1 | 0.987 | 2.752 | 0.002 | 15 | 0.003-0.999 |
| Plecoptera | Capniidae | Capnia | 1 | 0.618 | 1.722 | 0.002 | 1 | 0.005 |
| Araneae | Tetragnathidae | Tetragnatha | 1 | 0.927 | 2.583 | 0.003 | 13 | 0.001-0.947 |
| Diptera | Chaoboridae | Chaoborus | 1 | 0.655 | 1.825 | 0.003 | 3 | 0.005-0.962 |
| Diptera | Chironomidae | Endochironomus | 1 | 0.844 | 2.351 | 0.004 | 9 | 0.002-0.995 |
| Diptera | Chironomidae | Microchironomus | 1 | 0.720 | 2.005 | 0.03 | 51 | 0.001-0.995 |
| Diptera | Sciaridae | Bradysia | 1 | 0.625 | 1.742 | 0.046 | 21 | 0.001-0.611 |
|  |  | Residual | 144 | 51.675 |  |  |  |  |

Table S6. The five most variable bacterial ZOTUs in response to diet in *Sympetrum vulgatum* and *Lestes sponsa*. The distances to centroid derived from the constrained analysis of principal coordinates.

| Predator | ZOTU | Distance to centroid |
| --- | --- | --- |
| *S. vulgatum* | 1. Klebsiella Zotu1 | 1.762 |
|  | 2. Enterobacteriaceae Zotu2 | 1.294 |
|  | 3. Lactobacillales Zotu6 | 0.621 |
|  | 4. Hafnia Zotu4 | 0.395 |
|  | 5. Raoultella Zotu7 | 0.352 |
| *L. sponsa* | 1. Enterobacteriaceae Zotu2 | 1.479 |
|  | 2. Serratia Zotu3 | 1.414 |
|  | 3. Klebsiella Zotu1 | 1.217 |
|  | 4. Enterobacteriaceae Zotu13 | 1.188 |
|  | 5. Hafnia Zotu4 | 0.444 |

**References:**

1.	Kaunisto KM, Roslin T, Forbes MR, Morrill A, Saaksjarvi IE, Puisto AIE, Lilley TM, Vesterinen EJ (2020) Threats from the air: Damselfly predation on diverse prey taxa. J Anim Ecol 89: 1365–1374. doi: 10.1111/1365-2656.13184

2.	Morrill A, Forbes M, Vesterinen E, Tamminen M, Sääksjärvi I, Kaunisto KM (2024) Molecular characterisation of faecal bacterial assemblages among four species of syntopic Odonates. Microb Ecol 87. doi: <https://doi.org/10.1007/s00248-023-02328-1>

3.	Folmer O, Black M, Hoeh W, Lutz R, Vrijenhoek R (1994) DNA primers for amplification of mitochondrial cytochrome c oxidase subunit I from diverse metazoan invertebrates. Mol Mar Biol Biotechnol 3: 294–299.

4.	Jusino MA, Banik MT, Palmer JM, Wray AK, Xiao L, Pelton E, Barber JR, Kawahara AY, Gratton C, Peery MZ, Lindner DL (2019) An improved method for utilizing high-throughput amplicon sequencing to determine the diets of insectivorous animals. Mol Ecol Resour 19: 176–190. doi: 10.1111/1755-0998.12951

5.	Leray M, Yang JY, Meyer CP, Mills SC, Agudelo N, Ranwez V, Boehm JT, Machida RJ (2013) A new versatile primer set targeting a short fragment of the mitochondrial COI region for metabarcoding metazoan diversity: application for characterizing coral reef fish gut contents. Front Zool 10: 34. doi: 10.1186/1742-9994-10-34

6.	Geller J, Meyer C, Parker M, Hawk H (2013) Redesign of PCR primers for mitochondrial cytochrome c oxidase subunit I for marine invertebrates and application in all-taxa biotic surveys. Mol Ecol Resour 13: 851–861. doi: 10.1111/1755-0998.12138

7.	Apprill A, McNally S, Parsons R, Weber L (2015) Minor revision to V4 region SSU rRNA 806R gene primer greatly increases detection of SAR11 bacterioplankton. Aquat Microb Ecol 75: 129–137. doi: 10.3354/ame01753

8.	Kankaanpää T, Vesterinen E, Hardwick B, Schmidt NM, Andersson T, Aspholm PE, Barrio IC, Beckers N, Bety J, Birkemoe T, DeSiervo M, Drotos KHI, Ehrich D, Gilg O, Gilg V, Hein N, Hoye TT, Jakobsen KM, Jodouin C, Jorna J, Kozlov MV, Kresse JC, Leandri-Breton DJ, Lecomte N, Loonen M, Marr P, Monckton SK, Olsen M, Otis JA, Pyle M, Roos RE, Raundrup K, Rozhkova D, Sabard B, Sokolov A, Sokolova N, Solecki AM, Urbanowicz C, Villeneuve C, Vyguzova E, Zverev V, Roslin T (2020) Parasitoids indicate major climate-induced shifts in arctic communities. Global Change Biol 26: 6276–6295. doi: 10.1111/gcb.15297

9.	Vesterinen EJ, Puisto AIE, Blomberg AS, Lilley TM (2018) Table for five, please: Dietary partitioning in boreal bats. Ecol Evol 8: 10914–10937. doi: 10.1002/ece3.4559

10.	Vesterinen EJ, Ruokolainen L, Wahlberg N, Pena C, Roslin T, Laine VN, Vasko V, Saaksjarvi IE, Norrdahl K, Lilley TM (2016) What you need is what you eat? Prey selection by the bat Myotis daubentonii. Mol Ecol 25: 1581–1594. doi: 10.1111/mec.13564

11.	Rognes T, Flouri T, Nichols B, Quince C, Mahe F (2016) VSEARCH: a versatile open source tool for metagenomics. Peerj 4. doi: 10.7717/peerj.2584

12.	Martin M (2017) Cutadapt removes adapter sequences from high-throughput sequencing reads. EMBnetjournal 17: 10–12. doi: <http://dx.doi.org/10.14806/ej.17.1.200>.

13.	Ratnasingham S, Hebert PDN (2007) BOLD: The Barcode of Life Data System (www.barcodinglife.org). Mol Ecol Notes 7: 355–364. doi: 10.1111/j.1471-8286.2007.01678.x

14.	Vesterinen EJ, Kaunisto KM, Lilley TM (2020) A global class reunion with multiple groups feasting on the declining insect smorgasbord. Scientific Reports 10. doi: 10.1038/s41598-020-73609-9

15.	Edgar RC (2016) SINTAX: a simple non-Bayesian taxonomy classifier for 16S and ITS sequences. bioRxiv: 074161. doi: 10.1101/074161

16.	RCore (2023) A Language and Environment for Statistical Computing, Vienna, Austria

17.	RStudio (2024) RStudio: Integrated Development for R. RStudio, Inc., Boston, MA.

18.	Roberts D (2023) labdsv: Ordination and Multivariate Analysis for Ecology.

19.	Oksanen J SG, Blanchet F, Kindt R, Legendre P, Minchin P, O'Hara R,, Solymos P SM, Szoecs E, Wagner H, Barbour M, Bedward M, Bolker B,, Borcard D CG, Chirico M, De Caceres M, Durand S, Evangelista H,, FitzJohn R FM, Furneaux B, Hannigan G, Hill M, Lahti L, McGlinn D,, Ouellette M RCE, Smith T, Stier A, Ter Braak C, Weedon J (2024) vegan: Community Ecology Package_. R package version 2.6-8. pp. <https://CRAN.R>–project.org/package=vegan.

20.	Kassambara A (2023) ggpubr: 'ggplot2' Based Publication Ready Plots.

21.	Kassambara A (2023) rstatix: Pipe-Friendly Framework for Basic Statistical Tests.

22.	Fox J, Weisberg S (2019) An R Companion to Applied Regression. vol. 3rd.
